# Apolipoprotein M promotes growth and inhibits apoptosis of colorectal cancer cells through upregulation of ribosomal protein S27a

**DOI:** 10.17179/excli2020-2867

**Published:** 2021-01-21

**Authors:** Qinfeng Mu, Guanghua Luo, Jiang Wei, Lu Zheng, Haitao Wang, Miaomei Yu, Ning Xu

**Affiliations:** 1 Clinical Medical Research Center, the Third Affiliated Hospital of Soochow University, Changzhou 213003, China; 2 Gastrointestinal surgery, the Third Affiliated Hospital of Soochow University, Changzhou 213003, China; 3 Section of Clinical Chemistry and Pharmacology, Institute of Laboratory Medicine, Lunds University, Lund S-22185, Sweden

**Keywords:** Apolipoprotein M, growth, apoptosis, colorectal cancer, GeneChip microarrays, ribosomal protein S27a

## Abstract

Colorectal cancer (CRC) is one of the frequent malignant tumors and has a high mortality-to-incidence ratio. Apolipoprotein M (ApoM), a lipoprotein superfamily member, is primarily bound to high-density lipoprotein (HDL) particles. Our previous studies opined that ApoM crucially modulates CRC progression, but its role in CRC has not been elucidated. Here, lentivirus infection technology was used to overexpress ApoM in Caco-2 cells. Cell growth, apoptosis as well as clone formation assays were performed to explore the biological influences of ApoM in Caco-2 cells. Differentially expressed genes were analyzed via GeneChip microarrays and Quantitative real-time PCR (qPCR) along with Western blotting were applied to verify the results. Ribosomal protein S27a (RPS27A) expression in CRC and tumor-adjacent tissues was detected by qPCR, and its correlation with clinicopathologic characteristics was explored. Our results showed that ApoM overexpression could promote Caco-2 cell proliferation and inhibit apoptosis. The microarray evaluation uncovered 2671 genes, which were differentially expressed, including RPS27A. The qPCR as well as the Western blotting data showed that ApoM overexpression significantly increased the expression of RPS27A. Moreover, RPS27A expression was remarkably higher in CRC tissues in contrast with the tumor-adjacent tissues and was positively correlated with the ApoM level in tumor tissues, and higher RPS27A expression was associated with smaller tumors and lower T stage. Functional recovery experiments indicated that knockdown of RPS27A counteracted the apoptosis inhibition and clone formation promotion induced by ApoM overexpression in Caco-2 cells. In conclusion, ApoM promotes CRC cell growth and inhibits apoptosis through upregulation of RPS27A.

## Introduction

Colorectal cancer (CRC) is the 3^rd^ most frequent cancer diagnosed in both men and women as well as the 2^nd^ leading cause of cancer-linked death in the United States and worldwide (Ferlay et al., 2019[6]; Siegel et al., 2019[30]). Current treatments for CRC include surgery and chemotherapy, and the prognosis of patients primarily depends on the stage of the tumor and the location of the polyps (Oruc and Kaplan, 2019[22]). Despite advances in diagnostic and therapeutic strategies, the impact of these treatments on cure and survival rates is still limited (Marcuello et al., 2019[17]). CRC can be triggered by a variety of factors, among which genetic along with the environmental factors play a primary role in its pathogenesis (Wong et al., 2019[38]). A review by Pakiet et al. suggests that alterations in lipid composition and metabolism may be closely related to CRC risk, development and progression (Pakiet et al., 2019[23]). ApoM (Apolipoprotein M) was first identified and characterized by Xu and Dahlbäck in 1999 and belongs to the lipocalin superfamily (Xu and Dahlbäck, 1999[39]). ApoM is predominantly correlated with HDL (high-density lipoprotein) particles in the plasma and is responsible for approximately 5 % of HDL, as well as an even smaller percentage (0.2 ~ 1 %) of LDL (low-density lipoprotein) particles (Yu et al., 2019[42]). ApoM is involved in lipid metabolism, and whether ApoM is related to the development of CRC is worth exploring. Calayir et al. documented that the expression of ApoM could be upregulated by the LXR agonist in human colorectal adenocarcinoma Caco-2 cells (Calayir et al., 2008[2]). Our previous study revealed that ApoM mRNA levels were remarkably elevated in CRC tissues of individuals with lymph node metastasis (Luo et al., 2010[15]). Moreover, ApoM significantly increased the vitamin D receptor (VDR) mRNA levels in human colorectal adenocarcinoma HT-29 cells (Yu et al., 2017[43]). These results imply that ApoM may play a vital role in the regulation of CRC progression. However, how ApoM might participate in CRC and the possible molecular mechanisms have not yet been revealed or fully explored.

Ribosomal protein S27a (RPS27A) is an ubiquitin (Ub) C-terminal extension protein that performs extraribosomal functions, including participating in ribosome biogenesis as well as post-translational modification (Redman and Rechsteiner, 1989[25]; Warner and McIntosh, 2009[36]). Apart from functions mentioned above, Wang et al. showed that RPS27A could facilitate proliferation, modulate cell cycle progression as well as suppress apoptosis in chronic myeloid leukemia (CML) cells (Wang et al., 2014[35]). Gunasekaran et al. found that RPS27A is downregulated in virus-induced hepatocellular carcinoma (HCC) patients and that its expression has a weak inverse relationship with overexpression of the multifunctional protein YB-1 in HCC tissues (Gunasekaran and Ganeshan, 2014[8]). Hong et al. chronicled that RSP27A could be a prospective target in EBV-infected LMP1-positive cancer cells (Hong et al., 2017[9]). Additionally, Wong et al. reported that overexpression of RPS27A in human CRC is an early growth response gene (Wong et al., 1993[37]). Accumulating studies indicate that RPS27A participates in regulation of the progression of numerous cancer types.

Herein, we investigated ApoM's role in Caco-2 cell proliferation and apoptosis. We analyzed differentially expressed genes before and after ApoM overexpression (OE) in Caco-2 cells using GeneChip microarrays and found that RPS27A was significantly upregulated. Moreover, we detected the expression of RPS27A in CRC tumor as well as the corresponding tumor-adjacent tissues and analyzed its association with clinicopathological characteristics. Then, we conducted functional recovery by knocking down RPS27A in Caco-2 cells to determine whether ApoM affects these functions through RPS27A and further studied the possible downstream signaling cascades.

## Materials and Methods

### Tissue sample collection

From February 2014 to April 2015, 46 cases of CRC tumor and corresponding tumor-adjacent tissue specimens that were surgically removed and pathologically confirmed in the Gastrointestinal Surgery of the Third Affiliated Hospital of Soochow University were collected. No study subject was given preoperative chemotherapy and/or radiotherapy and they all gave written informed consent prior to enrolment. After excision we snap-froze the samples in liquid nitrogen and kept them at -80 °C until further use.

### Cell culture

The Caco-2 human colorectal adenocarcinoma cell lines were supplied by the Type Culture Collection of the Chinese Academy of Sciences (Shanghai, China). The cells were grown in Minimum Essential Medium (MEM) (Gibco, Life Technologies, USA) in presence of 10 % FBS (Gibco, Life Technologies, Australia), 100 U/mL penicillin as well as 100 μg/mL streptomycin (Gibco, Life Technologies, USA). The cells were allowed to grow at 37 °C under humidified parameters with 5 % CO_2_. 

### Lentivirus infection and RNA interference

Both construction and synthesis of recombined ApoM overexpression (LV-GV365-ApoM-OE) and RSP27A RNA interference (RNAi) (LV-GV248-RPS27A-RNAi) vectors with their corresponding negative control (NC) vectors were accomplished by GeneChem Co., Ltd. (Shanghai, China). The RPS27A RNAi targets at sequence 5'-TGGCAAATGTTGTCTGACTTA-3', and the scramble sequence was 5'-TTCTCCGAACGTGTCACGT-3'. The Caco-2 cells were transduced with the lentiviral vectors as suggested by the manufacturer. Cells were plated (2×10^5^ cells/mL) into 6-well plates and grew for 24 h to 30 ~ 50 % confluence, then we replaced the medium with infection medium with the added lentiviral vectors at an appropriate MOI (multiplicity of infection). Cells showing green fluorescent protein (GFP) positively observed under a fluorescence microscope after 72 h are regarded as successfully infected. Quantitative real-time PCR (qPCR) as well as Western blotting assays were applied for evaluating the efficiency of ApoM OE and RSP27A KD.

### GeneChip microarrays

The Affymetrix GeneChip microarrays were conducted by GeneChem Co., Ltd. (Shanghai, China). Isolation of the total RNA from the ApoM-OE cells as well as NC cells was conducted and the RNA quality and integrity were analyzed by the Thermo Scientific NanoDrop 2000 spectrophotometer (Waltham, MA, USA). First and second strand cDNA synthesis, *in vitro* transcription for synthesis of the labeled cRNA, and cDNA purification were performed using a GeneChip WT PLUS Kit (Affymetrix, Santa Clara, CA, USA). The purified cDNA was then processed with a GeneChip Hybridization, Wash and Stain Kit (Affymetrix). Finally, images and raw data were scanned with a GeneChip Scanner 3000 (Affymetrix). The visualization, quantification as well as the gene expression were analyzed with the GENEPIX 3.0 software (AXON) (Union City, CA, USA). The screening criteria for the genes that were remarkably differentially expressed were *P *< 0.05 as well as |Fold Change| > 2.

### RNA isolation and real-time qPCR

Extraction of total RNA was by employing TRIzol reagent (Pufei Biotechnology, Shanghai, China). The RNA concentration as well as the purity were evaluated via determining the absorbance at 260/280 nm with a BioPhotometer spectrophotometer (Eppendorf, Germany). The synthesis of cDNA was completed by using M-MLV RT (Promega, United States) as instructed by the manufacturer. qPCR assays involving SYBR Green master mix Takara Biotechnology, Dalian, China) were conducted on the Agilent Technologies Stratagene Mx3000P system (United States). The comparative threshold cycle approach (2^-ΔCt^) was employed to determine the relative levels of the mRNAs (Schmittgen and Livak, 2008[28]). GAPDH served as the internal standard. The sequences of primer used are demonstrated in Table 1(Tab. 1).

### Cell proliferation assessment

Cell Counting Kit-8 (Dojindo, Kumamoto, Japan) was employed to analyze cell proliferation ability. We transferred 6×10^3^ cells per well into a 96-well plate, with 6 replicates for each group. After that, the CCK-8 reagent was introduced to wells at 24, 48 as well as 72 h post-infection (10 μL/well), and the cells were evaluated 3 h later. Subsequently, the optical density (OD) values of the samples were determined at 450 nm to determine the number of viable cells. The OD 450-fold represents the OD values at each of the time point relative to the average of 24 h, revealing fold changes in cell proliferation. The data are derived from three independent assays performed in triplicate.

### Clone formation assay

After 72 h of infection, we inoculated 5×10^2^ cells/well into 6-well plates and left them to grow for 10 d, with medium changed every 3 d. The Olympus fluorescence microscope (Tokyo, Japan) was employed to capture the photographs of the clones of cells before terminating cell culture and rinsed once using PBS (phosphate-buffered saline). Thereafter, 4 % paraformaldehyde was employed to fix the cells for 30 min and then rinsed once more with PBS, followed by staining with crystal violet (Sangon Biotech, Shanghai, China). After rinsing using distilled-deionized water and complete drying, pictures of the cell clones were taken by a digital camera and cell counting was conducted. All samples in all groups were prepared in triplicate.

### Flow cytometry assay

The cells from each group were inoculated (2×10^5^ cells/mL) into 6-well plates at 72 h pot-transfection and allowed to grow to a confluence of approximately 85 %. After collecting the adherent ones and those in supernatant, cells were then resuspended to reach concentration of 1×10^6^ cells per mL in 1× of the binding buffer solution. For double staining to evaluate apoptosis, cells were costained using Annexin V/Alexa Fluor 647 as well as propidium iodide (PI) for 15 min at room temperature (RT) using an Annexin V-Alexa Fluor 647 Apoptosis Detection Kit (Fcmacs, Nanjing, China). Thereafter, the BD Biosciences FACScan flow cytometer installed with the Cell Quest software (San Jose, CA, USA) was employed to analyze the samples. For the single staining method, Annexin V-APC was employed to stain the cells using an Annexin V Apoptosis Detection Kit APC (eBioscience, San Diego, CA, United States) according to instructions provided by the manufacturer. The Millipore Guava easyCyte HT system (Billerica, MA, United States) along with the Guava InCyte software (Millipore) were deployed for flow cytometry.

### Western blotting analysis

Ice-cold PBS was employed to rinse the cells twice after harvesting. After that, cell lysis was performed using the RIPA lysis buffer as described by the manufacturer (Beyotime, Shanghai, China). Then, we utilized the BCA protein assay kit to quantify the proteins (Beyotime). After 5 × SDS-PAGE loading buffer (Beyotime) was added and mixed, protein samples were boiled for 5 min. Protein with the amount of 30 μg was successfully fractionated with a 10 % SDS-PAGE gel and blotted onto a PVFD membrane (Merck Millipore, Billerica, MA, USA). Subsequently, 3 % TBST solution containing 5 % dry milk was used to block the membranes at RT for 1 h. After that, we inoculated the membranes with anti-ApoM (F2051-1G9, Abnova, Taipei, Taiwan) and anti-RPS27A (#3936, Cell Signaling Technology, Boston, MA, USA) antibodies at 4 °C overnight. GAPDH (sc-32233, Santa Cruz, CA, USA) was employed as the standard for normalizing protein loading. After rinsing four times using TBST, the membranes were probed with HRP-conjugated AffiniPure goat anti-rabbit IgG (#7074, CST) or horse anti-mouse IgG (#7076, CST) at RT for 1.5 h. Afterwards, the Pierce ECL Western Blotting Substrate kit (Thermo Scientific, Rockford, IL, United States) was used to visualize protein bands after washing the membranes with TBST.

### Statistical analyses

The GraphPad Prism 6.0 software (Inc, San Diego, CA, USA) was applied for statistical analyses. The data are indicated as the means ± SD. A Student's *t* test (two-tailed) was employed to evaluate the significant differences between the two groups. A Pearson correlation test was utilized for correlation analysis. One-way analysis of variance (ANOVA), and subsequently Dunn's multiple comparison test were performed for multiple comparisons. *P* < 0.05 signified statistical significance.

## Results

### Effect of ApoM on Caco-2 cell proliferation and apoptosis

**Figure 1:**
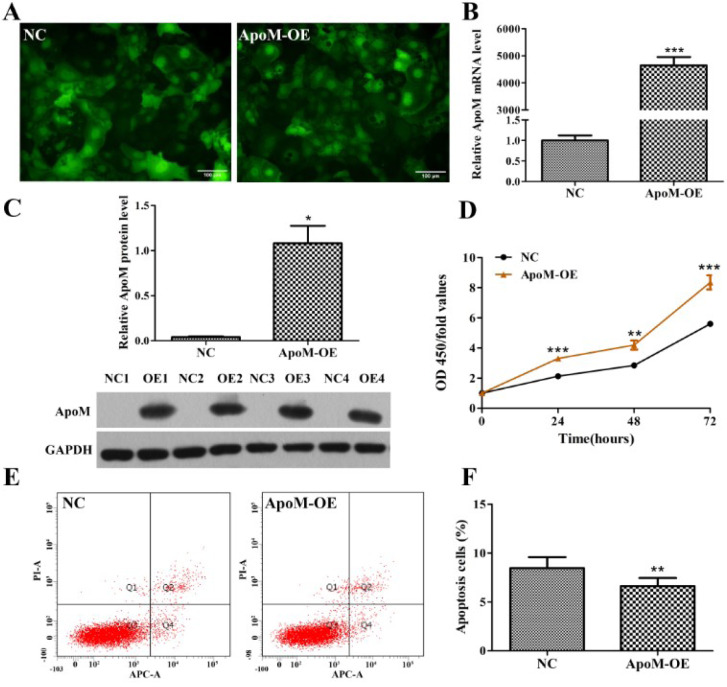
Effect of ApoM on Caco-2 cell proliferation and apoptosis. (A) Fluorescence micrographs of Caco-2 cells taken 72 h after infection. Scale bar, 100 μm. (B) The relative ApoM mRNA level in Caco-2 cells was determined by qPCR and normalized to the GAPDH level. Fold change was calculated using 2^−ΔCt^. (C) The ApoM protein level in Caco-2 cells was determined by Western blotting. Both the ApoM-OE and NC groups include four replicates. GAPDH was the loading control. (D) Cell proliferation was measured using CCK-8 assays. OD values were measured at 450 nm. Cell growth curves were plotted based on OD 450-fold values at different time points. (E) Apoptotic cells were costained with Annexin V-APC and PI and measured using flow cytometry. Q1 represents necrotic cells, Q2 represents late apoptosis cells, Q3 represents viable cells, and Q4 represents early apoptotic cells. Apoptosis cells are present in Q2+Q4. (F) The Caco-2 cell apoptosis rate in the ApoM-OE group was lower than in the NC group. The data represent three independent experiments performed in triplicate and are presented as the means ± SD. Student's *t* test was used to analyze significant differences: ^*^P < 0.05, ^**^P < 0.01, and ^***^P < 0.001 versus NC. ApoM, apolipoprotein M; qPCR, quantitative PCR; CCK, cell counting kit; OD, optical density; APC, allophycocyanin; PI, propidium iodide; OE, overexpression; NC, negative control.

Caco-2 cells successfully infected with lentivirus could express GFP. After 72 h of infection, the inverted fluorescence microscope observation revealed green fluorescence. As manifested in Figure 1A(Fig. 1), the proportion of GFP-positive cells in both the ApoM-OE and NC group was over 90 %. The overexpression efficiency of ApoM was evaluated via qPCR along with Western blotting. Consequently, the qPCR data indicated that the ApoM mRNA level in the ApoM-OE group was increased to more than 4,000 times relative to the NC group (*P *< 0.0001, Figure 1B(Fig. 1)). Similarly, the Western blotting analysis demonstrated that the ApoM protein level in the ApoM-OE group was remarkably elevated (*P *< 0.05, Figure 1C(Fig. 1)).

To investigate whether ApoM influences the biological function of Caco-2 cells, CCK-8 and the flow cytometry assays were employed to assess cell proliferation and apoptosis, respectively. As revealed by the CCK-8 results, the OD 450-fold values in the ApoM-OE group at 24, 48 as well as 72 h post-infection were remarkably higher in contrast with those in the NC group as indicated in Figure 1D(Fig. 1). The flow cytometry data demonstrated that rate of apoptosis of the Caco-2 cells in the ApoM-OE group was remarkably lower relative to the NC group (*P *< 0.01, Figure 1E and 1F(Fig. 1)).

### RPS27A was significantly upregulated in ApoM-OE Caco-2 cells

**Figure 2:**
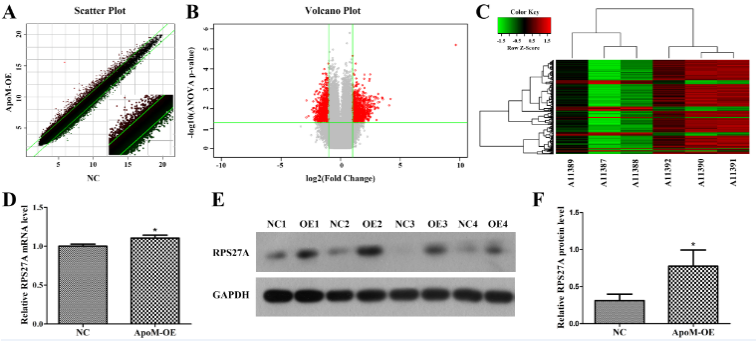
Differentially expressed genes between the ApoM-OE and NC groups. (A) Scatter plot. The X-axis represents the NC group, and the Y-axis represents the ApoM-OE group. (B) Volcano plot. The X-axis represents the logarithm conversion of the fold difference to base 2, and the Y-axis represents the logarithm conversion of the corrected significant levels to base 10. Red indicates all the probes with |Fold Change| > 2 and *P *< 0.05. (C) Heat map of hierarchical clustering. Each column represents a sample, and each row represents a differentially expressed gene. The upper dendritic structure indicates the aggregation or classification of all samples; the left dendritic structure indicates the aggregation of expression patterns of differentially expressed genes. (D) The relative RPS27A mRNA level in Caco-2 cells was determined by qPCR. (E) The RPS27A protein level in Caco-2 cells was determined by Western blotting. Both the ApoM-OE and NC groups include four replicates. GAPDH was the loading control. (F) The relative RPS27A protein level was significantly higher in the ApoM-OE group than in the NC group. The data are presented as the means ± SD. Student's *t* test was used to analyze significant differences; ^*^P < 0.05 versus NC. ApoM, apolipoprotein M; qPCR, quantitative PCR; RPS27A, ribosomal protein S27a; OE, overexpression; NC, negative control.

We utilized the GeneChip microarrays to compare gene expression profiles between the Caco-2 cell ApoM-OE and NC groups. After verifying the quality of the microarray data, we performed a significant difference analysis. In Figure 2A(Fig. 2), the scatter plot illustrates the distribution of the signal strength between the ApoM-OE and NC groups on a Cartesian coordinate plane. The two parallel green lines were used as differential reference lines, and the points between them represent genes with no remarkable changes. Besides, the red dots outside the interval indicate the relatively upregulated probes in the ApoM-OE group, and the green dots indicate the relatively upregulated probes in the NC group. A volcano plot was drawn to show the distribution of the genes that were differentially expressed between the ApoM-OE and NC groups. The red dots represent all genes remarkably differentially expressed screened by |Fold Change| > 2 and *P *< 0.05, and the gray dots were genes with no significant expression differences (Figure 2B(Fig. 2)). Figure 2C(Fig. 2) presents a heat map of hierarchical clustering of ApoM-OE and NC samples according to the expression profiles of the screened differential genes. Red reveals that the expression of the gene was relatively upregulated, green indicates downregulated, black designates no remarkable change, and gray designates that the signal intensity of the gene was not detected. Based on the above screening criteria, a total of 1324 upregulated genes and 1347 downregulated genes were uncovered in the ApoM-OE group in contrast with the NC group. For instance, the upregulated genes included RPS27A, ubiquitously expressed prefoldin-like chaperone (UXT), apolipoprotein C1 (APOC1) and APOM, and the downregulated genes included MDM4 regulator of p53 (MDM4), natural killer cell triggering receptor (NKTR), vascular endothelial growth factor A (VEGFA) and LDL receptor-related protein 6 (LRP6) (Table 2(Tab. 2)). We further verified the expression level of the representative upregulated gene RPS27A using qPCR and Western blotting. The qPCR data indicated that the expression level of RPS27A mRNA in the ApoM-OE Caco-2 cells was remarkably higher in contrast with that in the NC group (*P *< 0.05, Figure 2D(Fig. 2)). Besides, in contrast with the NC group, the protein expression level of RPS27A in the ApoM-OE group Caco-2 cells was remarkably increased (*P *< 0.05, Figure 2E and 2F(Fig. 2)).

### RPS27A was significantly upregulated in CRC patients

**Figure 3:**
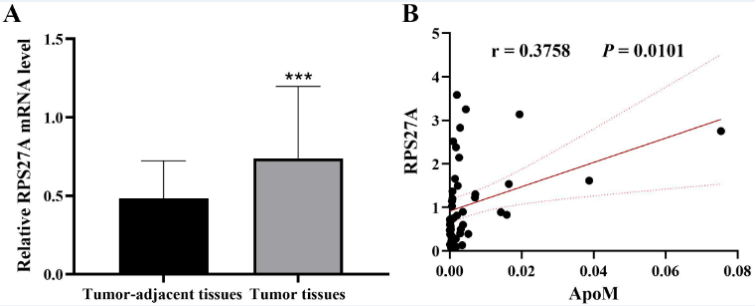
RPS27A expression in CRC tissues. (A) RPS27A expression in tumors and corresponding tumor-adjacent tissue specimens from 46 CRC cases was quantified via qPCR and normalized to GAPDH expression. Fold-change was calculated using 2^-ΔCt^. The data are compared using a Student's *t* test and are shown as the means ± SD; ^***^P < 0.001. (B) The correlation between RPS27A expression and ApoM expression in CRC tissues was analyzed using the Pearson correlation method, and r is defined as the Pearson correlation coefficient. CRC, colorectal cancer; RPS27A, ribosomal protein S27a; qPCR, quantitative PCR; NC, negative control. ApoM affects the biological roles of Caco-2 cells through RPS27A

RPS27A expression was measured in tumor and corresponding tumor-adjacent tissue specimens from 46 CRC cases using qPCR. The data demonstrated that RPS27A in tumor tissues was remarkably higher relative to that in tumor-adjacent tissues (*P *< 0.0001, Figure 3A(Fig. 3)). Pearson correlation analysis demonstrated that RPS27A expression was positively linked to the ApoM level in tumor tissues (r = 0.3758, *P *= 0.0101, Figure 3B(Fig. 3)). Analysis of clinicopathological parameters illustrated that the expression of RPS27A in tumor tissues ≥ 6.0 cm was remarkably lower in contrast with that in tumor tissues < 6.0 cm and was remarkably lower in T3/4 stage patients in contrast with that in T1/2 stage patients (*P *< 0.01). As shown in Table 3(Tab. 3), there was no remarkable difference in gender, age, N/M stage or lymph node metastasis.

**Figure 4:**
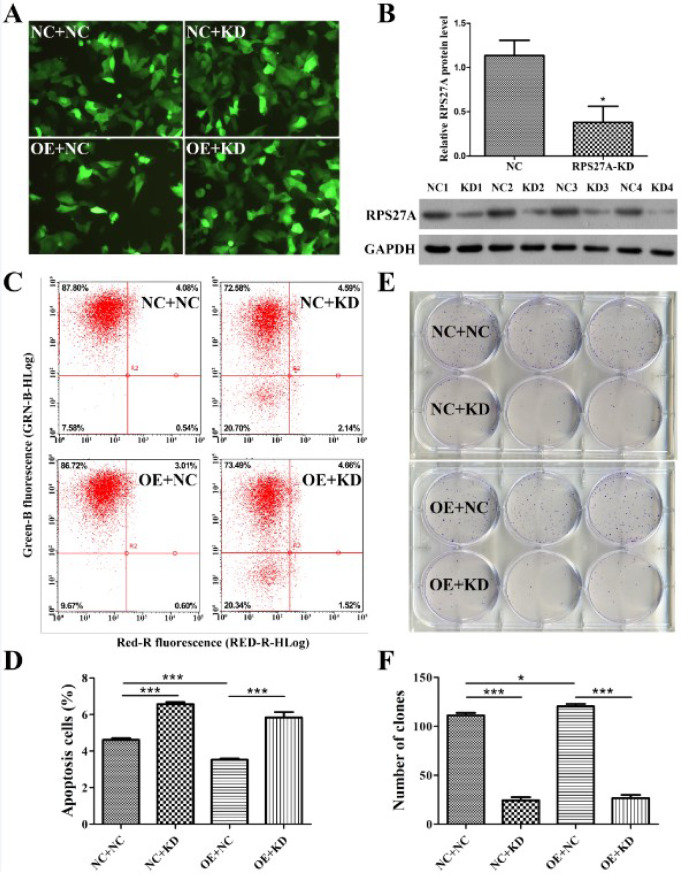
ApoM affects the biological functions of Caco-2 cells through RPS27A. (A) Fluorescence micrographs of Caco-2 cells in each group taken 72 h after infection with the RSP27A RNAi lentiviral vector. Scale bar, 100 μm. (B) The RPS27A protein level in Caco-2 cells was determined by Western blotting. Both the RSP27A-KD and NC groups include four replicates. GAPDH was used as the internal reference. (C) Apoptotic cells were stained with Annexin V-APC and measured using flow cytometry. The abscissa represents red fluorescence (RED-R-HLog), and the ordinate represents green fluorescence (GRN-B-HLog). (D) Statistical analysis was performed to determine the percentage of apoptotic cells in each group. (E) Cell clones were stained with crystal violet and photographed with a digital camera. Triplicate samples were prepared for each experimental group. (F) The number of clones was accurately calculated and statistically analyzed. Each experimental group was prepared in triplicate. The data are presented as the means ± SD. Student's *t* test was used to analyze significant differences; ^*^P < 0.05 and ^***^P < 0.001 versus NC. ApoM, apolipoprotein M; qPCR, quantitative PCR; RPS27A, ribosomal protein S27a; OE, overexpression; APC, allophycocyanin; NC, negative control

To examine further the mechanism underlying the biological role of ApoM in CRC, ApoM overexpression (ApoM-OE) as well as negative control (ApoM-NC) Caco-2 cells were stably transduced with the RPS27A RNAi (RSP27A-KD) lentiviral vector and its negative control vector (RSP27A-NC), respectively. In this experiment, we used ApoM-OE and its control lentiviral vector without GFP, while RPS27A RNAi lentiviral vector and its control vector could induce GFP expression. As shown in Figure 4A(Fig. 4), the NC+NC group represents ApoM-NC Caco-2 cells infected with RSP27A-NC lentiviral vector; NC+KD group represents ApoM-NC cells infected with RSP27A-KD lentivirus; OE+NC group represents ApoM-OE cells infected with RSP27A-NC lentivirus; OE+KD group represents ApoM-OE cells infected with RSP27A-KD lentivirus. The percentage of GFP-positive cells in each group was over 90 %. According to Western blotting results, the RPS27A protein level was remarkably decreased after RNAi-mediated knockdown in Caco-2 cells (*P *< 0.05) as indicated in Figure 4B(Fig. 4). The flow cytometry data demonstrated that the rate of apoptosis of Caco-2 cells in the OE+NC group was remarkably lower in contrast with that of cells in the NC+NC group. Moreover, the rates of apoptosis in the NC+KD and OE+KD group were higher in contrast with those in their respective control groups (*P *< 0.001, Figure 4C and 4D(Fig. 4)). Conversely, the Caco-2 cell clone formation assay showed that the number of clones in the OE+NC group was remarkably higher relative to that in the NC+NC group (*P *< 0.05), whereas the number of clones in the NC+KD and OE+KD groups was lower than in their respective control groups (*P *< 0.001, Figure 4E and 4F(Fig. 4)).

## Discussion

CRC is one of the most common malignant tumors and is associated with high incidence and mortality rates (Siegel et al., 2017[29]). Abnormal levels of lipids, particularly HDL and LDL, and lipid metabolism have been linked with cancer risk and progression in some malignancies (Sirnio et al., 2017[31]). Apolipoproteins, an important component of lipoprotein HDL and LDL complexes, are involved in lipid metabolism regulation. High serum Apolipoprotein A1 (APOA1) and HDL levels have been reported to be associated with a reduced risk of colorectal adenomas and CRC (Coppola et al., 2015[5]; Tian et al., 2015[34]). Our previous studies showed that both ApoM mRNA and protein expression levels were significantly lower in CRC tissues than in normal and benign colorectal tissues (Mu et al., 2012[18]). Moreover, ApoM mRNA levels in CRC tissues were significantly increased in the patients with lymph node metastasis (Luo et al., 2010[15]). In this study, we constructed ApoM-overexpressing lentiviral vectors and successfully infected Caco-2 cells. The qPCR and Western blotting results showed that ApoM mRNA and protein levels were significantly increased in ApoM-overexpressing Caco-2 cells. On this basis, we systematically evaluated the biological function of ApoM in CRC cells. The results showed that ApoM overexpression significantly promoted Caco-2 cell proliferation and inhibited apoptosis, suggesting that ApoM might function as an oncogene in CRC development and progression. 

Microarray analyses revealed a total of 2671 differentially expressed genes, including 1324 upregulated and 1347 downregulated genes. Partial differentially expressed genes between the ApoM-OE and NC group are shown in Table 2(Tab. 2). Among these genes, MDM4 has been reported to play an important role in negative regulation of the tumor suppressor p53 through its interaction with MDM2 (Gansmo et al., 2015[7]). MDM4/ MDM2 double knockdown with siRNAs enhanced 5-fluorouracil (5-FU)-induced p53 activation, arrested cell cycle at G1 phase, and potentiated the antitumor effect of 5-FU in wtTP53/highMDM4 human colon cancer cells (Imanishi et al., 2019[10]). Suda et al. showed that MDM4 was abnormally expressed in CRC tumor tissues compared with levels in matched normal mucosa and speculated that MDM4 might play a role in colorectal carcinogenesis (Suda et al., 2011[32]). Natural killer (NK) cells contribute to the first line of defense against cancer and virus infection as critical components of the innate immune system (Li et al., 2018[14]). NKTR is present on the surface of NK cells and acts as a part of the putative NK target recognition complex that facilitates binding to targets (Cantoni et al., 1999[3]; Ponassi et al., 2003[24]). Krijgsman et al. reported that the expression levels of the natural cytotoxic receptors NKp44 and NKp46 on both CD56dim NK cells and NKT-like cells were significantly reduced in CRC patients compared with healthy donors (Krijgsman et al., 2019[12]). Vascular endothelial growth factor (VEGF) is one of the most important and specific factors that stimulate both physiological and pathological angiogenesis (Lan et al., 2017[13]). VEGFA, which is often referred to as VEGF, has been found to be overexpressed in CRC, and its overexpression was identified as a marker of poor patient survival (Mammadova-Bach et al., 2018[16]). LDL receptor-related protein-6 (LRP6), an important coreceptor in the Wnt pathway, is significantly upregulated at the transcriptional, protein and phosphorylation levels in CRC cells and tissues and is correlated with TNM or Dukes staging and a worse prognosis (Rismani et al., 2017[27]; Yao et al., 2017[40]). The long noncoding RNA UXT antisense 1 (UXT-AS1) was found to be significantly upregulated in CRC, and high expression levels of UXT-AS1 were significantly associated with a poor prognosis in CRC patients. Moreover, aberrantly high UXT-AS1 expression was found to promote CRC progression by changing the alternative splicing of UXT from the UXT1 transcript to the UXT2 transcript (Yin et al., 2017[41]). Apolipoprotein C1 (APOC1) belongs to the apolipoprotein C family and is a secreted protein involved in lipoprotein metabolism. Recent studies have shown that APOC1 is overexpressed in CRC tissues and that a high APOC1 level contributes to a poor prognosis. APOC1 expression influenced the proliferation ability and motility capacity of CRC cells via the MAPK pathway (Ren et al., 2019[26]). In addition, RPS27A (also known as UBC, S27A, CEP80, UBA80, HEL112, UBCEP1, and UBCEP80) is an extension protein on the carboxy terminus of ubiquitin (Kirschner and Stratakis, 2000[11]) and has been reported to be an early growth response gene overexpressed in human CRC (Barnard et al., 1995[1]; Wong et al., 1993[37]). Thus, these differentially expressed genes are closely related to the occurrence and development of CRC. Based on the function of these genes, we deduced that ApoM might participate in CRC progression by affecting the expression of these genes. 

Furthermore, we examined the expression of RPS27A in tumor and corresponding tumor-adjacent tissue specimens from 46 CRC cases and found that RPS27A expression in tumor tissues was significantly higher than in tumor-adjacent tissues. Pearson correlation analysis showed that RPS27A expression was positively correlated with ApoM expression in CRC. From this result, we conclude that RPS27A is involved in the CRC process and may act as an oncogene. Table 3(Tab. 3) shows that higher RPS27A expression was associated with smaller tumors and lower T stage, which seems to contradict the hypothesis that RPS27A plays a pro-cancer role in CRC. The possible reason is that RPS27A might participate in feedback regulation mechanisms in CRC. According to a previous report, living systems can maintain homeostasis and adapt to constantly challenging environmental conditions through feedback controls that operate at multiple levels in cells and tissues. Feedback regulation of signal transduction operates on distinct timescales through protein-protein interactions (PPIs) and post-translational modifications (PTMs) (Nguyen and Kholodenko, 2016[20]). Muller-McNicoll et al. (2019[19]) reported that the autoregulatory feedback to translation by ribosomal proteins (r-proteins) is not restricted to bacteria but can also be detected in eukaryotes, for example, yeast RPS9 and RPS28 and human RPS13. Based on this, we infer that when tumor volume increases and adjacent tissues are affected in CRC and as RPS27A protein accumulates to higher concentrations, negative feedback will be triggered: RPS27A binds to regulatory sequences present in its own mRNA and exerts its autoregulatory function, which limits further protein synthesis. This might explain the lower RPS27A expression in tissues of CRC patients with larger tumors and higher T grade. In addition, a larger sample size may be more representative and persuasive, and we will expand the sample size and conduct detailed analyses in the future.

Further qPCR and Western blotting results showed that ApoM overexpression significantly increased the expression level of RPS27A, which was consistent with the microarray data. Subsequently, functional recovery experiments indicated that RPS27A interference counteracted the inhibition of cell apoptosis and promotion of cell clone formation induced by ApoM overexpression in Caco-2 cells. *In* *vivo* and *in* *vitro* studies have shown that RPS27A can promote cell proliferation and invasion, regulate cell cycle progression and inhibit apoptosis through a variety of mechanisms. RPS27A was reported to interact with MDM2 and suppress MDM2-mediated p53 ubiquitination, leading to p53 activation and cell cycle arrest. In turn, MDM2 ubiquitinates RPS27A and promotes proteasomal degradation of RPS27A in response to ribosomal stress, thus forming a mutual-regulatory loop (Sun et al., 2011[33]). Based on the study by Nosrati et al., we clearly recognized that RPS27A is both an inducer of p53 under nucleolar stress and a target of p53 under DNA damage conditions. RPS27A is a crucial molecular sensor that must be maintained at optimal levels for normal cell cycle progression, and both its overexpression and knockdown can result in activation of cell cycle inhibitors, such as p21^Waf1^ (Nosrati et al., 2015[21]). Similarly, the findings of Chen et al. suggest that nicotine promotes human papillomavirus (HPV)-immortalized cervical epithelial (H8) cell proliferation by activating the RPS27A-MDM2-p53 pathway *in vitro *(Chen et al., 2019[4]). Hence, it was speculated that ApoM regulates the expression of RPS27A and might affect the biological function of CRC cells through the MDM2-p53 pathway. However, the specific mechanisms have yet to be fully elucidated.

In conclusion, ApoM promotes CRC Caco-2 cell growth and inhibits apoptosis through upregulation of RPS27A.

## Notes

Miaomei Yu and Ning Xu (Section of Clinical Chemistry and Pharmacology, Institute of Laboratory Medicine, Lunds University, Klinikgatan 19, Lund S-22185, Sweden; E-mail: ning.xu@med.lu.se) contributed equally as corresponding authors.

## Declaration of competing interest

The authors declare that they have no conflicts of interest.

## Funding

This work was supported by the Changzhou Applied Basic Research Project (No: CJ20190092), funding from Young Talent Development Plan of Changzhou Health Commission (2020-233), the International Cooperation Item of Changzhou (No. CZ20190022) and Special Program for Foreign High-Level Talents Introduction (CQ20204035).

## Authors' contributions

QFM, MMY and NX designed the research; QFM, GHL, JW and HTW performed the experiments; LZ analyzed the data; QFM produced the figures; QFM wrote the manuscript; MMY, NX revised the manuscript.

## Figures and Tables

**Table 1:**
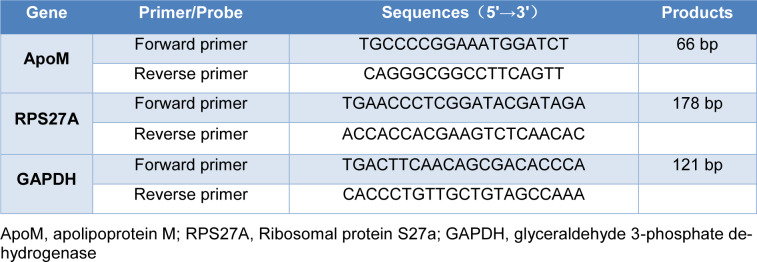
Sequences of primers

**Table 2:**
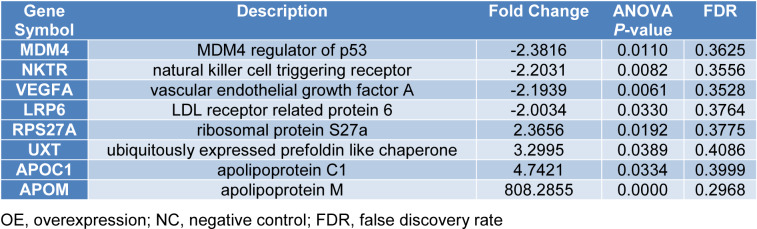
Partial differentially expressed genes between ApoM-OE and NC group

**Table 3:**
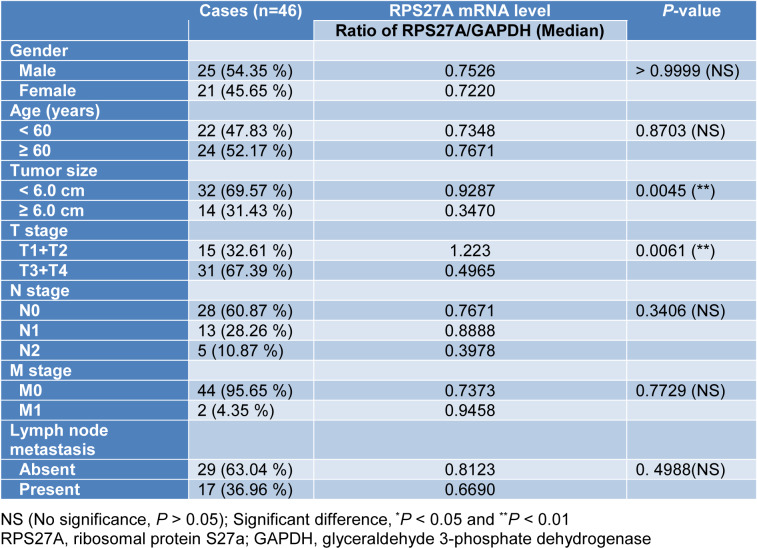
Association of RPS27A expression with clinicopathological parameters
